# Curcumin’s Metabolites, Tetrahydrocurcumin and Octahydrocurcumin, Possess Superior Anti-inflammatory Effects *in vivo* Through Suppression of TAK1-NF-κB Pathway

**DOI:** 10.3389/fphar.2018.01181

**Published:** 2018-10-17

**Authors:** Zhen-Biao Zhang, Dan-Dan Luo, Jian-Hui Xie, Yan-Fang Xian, Zheng-Quan Lai, Yu-Hong Liu, Wei-Hai Liu, Jian-Nan Chen, Xiao-Ping Lai, Zhi-Xiu Lin, Zi-Ren Su

**Affiliations:** ^1^Guangdong Provincial Key Laboratory of New Drug Development and Research of Chinese Medicine, Mathematical Engineering Academy of Chinese Medicine, Guangzhou University of Chinese Medicine, Guangzhou, China; ^2^School of Chinese Medicine, Faculty of Medicine, The Chinese University of Hong Kong, Hong Kong, China; ^3^Guangdong Provincial Key Laboratory of Clinical Research on Traditional Chinese Medicine Syndrome, The Second Affiliated Hospital, Guangzhou University of Chinese Medicine, Guangzhou, China; ^4^Department of Pharmacy, Shenzhen University General Hospital, Shenzhen University, Shenzhen, China; ^5^Guangdong Food and Drug Vocational College, Guangzhou, China; ^6^Higher Education Institute and Development Research of Chinese Medicine, Guangzhou University of Chinese Medicine, Guangzhou, China; ^7^Dongguan Mathematical Engineering Academy of Chinese Medicine, Guangzhou University of Chinese Medicine, Dongguan, China

**Keywords:** tetrahydrocurcumin, octahydrocucumin, curcumin, inflammation, COX-2, TAK1-NF-κB pathway

## Abstract

Curcumin (CUR), a promising naturally occurring dietary compound, is commonly recognized as the potential anti-inflammatory agent. While the application of CUR was hampered by its low stability and poor systemic bioavailability, it has been suggested that the biological activities of CUR are intimately related to its metabolites. In the current investigation, we aimed to comparatively explore the anti-inflammatory effects of tetrahydrocurcumin (THC), octahydrocurcumin (OHC), and CUR, and to elucidate the underlying action mechanisms on experimental mice models of acute inflammation, i.e., xylene-induced ear edema, acetic acid-induced vascular permeability, and carrageenan-induced paw edema. The results showed that THC and OHC exerted significant and dose-dependent inhibitions on the formation of ear edema induced by xylene and paw edema provoked by carrageenan and inhibited the Evans blue dye leakage in peritoneal cavity elicited by acetic acid. Moreover, THC and OHC treatments were more effective than CUR in selectively inhibiting the expression of cyclooxygenase 2 (COX-2) and suppressing nuclear factor-κB (NF-κB) pathways via transforming growth factor β activated kinase-1 (TAK1) inactivation in the carrageenan-induced mouse paw edema model.

## Introduction

**FIGURE 1:**
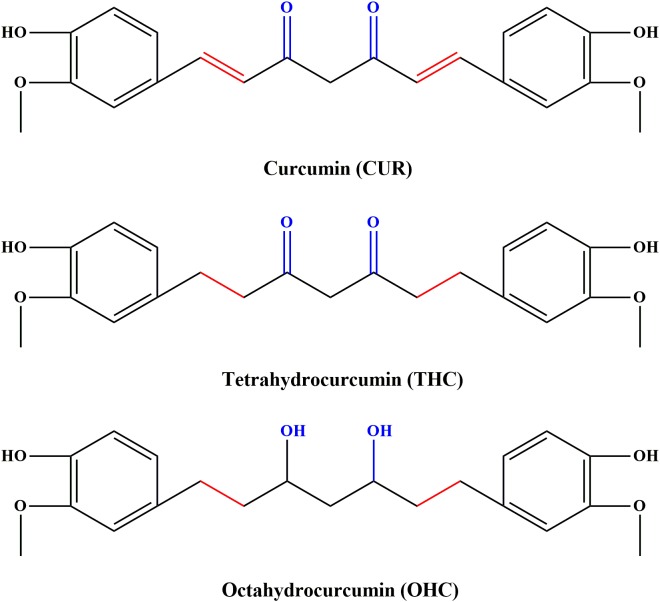
Chemical structures of curcumin (CUR), tetrahydrocurcumin (THC), and octahydrocucumin (OHC).

Curcumin (CUR, C_21_H_20_O_6_, the molecular structure depicted in **Figure 1**), a food pigment derived from the curcuma species like dietary spice turmeric (rhizome of *Curcuma longa*), has been commonly used as a flavoring agent in various foods. Over the past decades, CUR has received an increasing research attention, largely due to its diverse biological properties including anti-inflammatory, anti-cancer, anti-oxidant and anti-bacterial activities (Pan et al., 2000; Sandur et al., 2007; Agrawal and Mishra, 2010). However, recent studies indicated that application of CUR was hindered by its poor systemic bioavailability (Anand et al., 2007). For instance, the levels of CUR were found to be very low in serum and tissues even for high dose of exposure (Sharma et al., 2001). The deficiency seriously hinders its clinical application. It has been found that oral administration with CUR in mice resulted in reduction to dihydrocurcumin, tetrahydrocurcumin (THC, C_21_H_24_O_6_, the chemical structure shown in **Figure 1**) and hexahydrocurcumin mediated by endogenous reductase (Zhongfa et al., 2012). THC has been characterized to be the primary metabolite responsible for diverse biological properties (Lee et al., 2005), including anti-inflammation (Zhao et al., 2015). In addition, previous studies have demonstrated that octahydrocurcumin (OHC, C_21_H_28_O_6_, the chemical structure shown in **Figure 1**), another important metabolite of CUR, exerted appreciable anti-inflammatory properties in lipopolysaccharide (LPS)-challenged RAW 264.7 murine macrophages model (Zhao et al., 2015). However, the *in vivo* anti-inflammatory effects of THC and OHC have not been explored so far. Despite OHC has been discovered in earlier studies, difficulties in its availability has hampered the studies on its biological properties. To solve this predicament, our previous study has developed a simple and highly effective way to produce OHC (Zhang et al., 2018), which made it possible to explore its biological activities.

Inflammation is the key biological process for eliminating the initial cause of cell injury in the host defense system (Huang et al., 2010). However, inflammatory process can cause tissue injuries, leading to various disorders, such as inflammatory bowel disease and rheumatoid arthritis (Monaco et al., 2004; Akaogi et al., 2006). Extensive effort has been devoted to the exploration of anti-inflammatory agents, and suppression of synthesis of prostaglandins (PGs) is the focus of present anti-inflammatory research. Cyclooxygenase (COX) plays an important role in the synthesis of PGs from arachidonic acid (Vane, 1971) and there are two subtypes of COX, namely, COX-1 and COX-2 (Roth et al., 1975). COX-1 predominantly acts as a housekeeper in various biological processes, including gastrointestinal (GI) mucosa protection, and depression of COX-1 could result in GI tract damage (Radi and Khan, 2006). By contrast, COX-2 exhibits a low level of expression or is even absent in most normal tissues. However, COX-2 is inducible and up-regulated by inflammatory processes upon the presence of inflammatory or tissue-injury stimulus, such as tumor necrosis factor-α (TNF-α), interleukin-1β (IL-1β) and IL-6, and could conversely promote the development of inflammation (Patrignani et al., 1994; Ivanov et al., 2002). In addition, COX-2-mediated prostaglandinE2 (PGE_2_) production could also worsen inflammatory diseases. Taken together, highly selective COX-2 inhibitors are extremely desirable for the treatment of inflammatory diseases.

Recent studies have demonstrated that transforming growth factor β activated kinase-1 (TAK1) performs an essential role in activating the TNF-α and IL-1β-mediated nuclear factor-κB (NF-κB) signaling pathway (Doyle and O’Neill, 2006). As a member of the MAP3K family (Yamaguchi et al., 1995), there are many regulatory subunits that could affect TAK1 activity, such as TAK1-binding protein 1 (TAB1), TAB2 and TAB3 (Prickett et al., 2008). Under physiological conditions, TAK1 binds to TAB1, and subsequently forms a complex of TAK1/TAB1/TAB2 in the membrane in the presence of TAB2 (Doyle and O’Neill, 2006). Once phosphorylated TAK1 (p-TAK1) escapes from the cell membrane, the activation of κBs kinase inhibitor (IκBs), and downstream targets such as IKKs will then be phosphorylated (Shim et al., 2005). After activation, IKKs would result in the phosphorylation and degradation of IκBs and further to motivate the NF-κB pathway (Hacker and Karin, 2006). Based on the previous studies on the relationship between CUR and NF-κB (Santos et al., 2014), in the present study, we focused on: (1) comparatively explore the *in vivo* anti-inflammatory effect of THC and OHC with CUR using three common inflammatory animal models; (2) probe into the molecular mechanisms.

## Materials and Methods

### Materials and Reagents

CUR, THC, Tween-80, and carrageenan were purchased from the Sigma-Aldrich (St. Louis, MO, United States). OHC was obtained in our laboratory. Evans blue and xylene were obtained from Sinopharm (Shanghai, China). Indomethacin (Indo) was purchased from Huanan Pharmacy Company (Guangzhou, China). The purity of CUR and THC was found to be >99.4%, and the purity of OHC was found to be >98.06% as determined by HPLC. All the reagents and chemicals employed in the current study were at least of analytical grade.

### Animals

Male and female ICR mice weighing 18–22 g were obtained from Guangdong Medical Laboratory Animal Center (Foshan, China). All animals were housed under regulated temperature (22 ± 2°C), humidity (50 ± 10%) with a 12-h light/ dark cycle and free access to forage and clean water. This study was carried out in accordance with the National Institutes of Health (NIH) guidelines for the Care and Use of Laboratory Animals. The protocol was approved by the Animal Experimental Ethics Committee of Guangzhou University of Chinese Medicine (Guangzhou, China, No. 2017056). Mice were acclimatized to the laboratory conditions for 7 days prior to the experiment. With the exception of acute toxicity experiment, animals of either sex were randomly divided to 10 groups (*n* = 10), of which the intact and vehicle controls were intragastrically (i.g.) treated with 0.1% Tween-80 throughout the experiments. Other groups were treated (i.g.) with different concentrations of THC, OHC (10, 20, and 40 mg/kg), CUR (100 mg/kg), and indomethacin (Indo, 10 mg/kg, reference drug), respectively, for 7 days. All the concentrations of agents were based on the results of the preliminary experiments (**Supplementary Figure S1**). All agents were dissolved in 0.1% Tween-80 and treated once every day.

### Acute Toxicity Test

The median lethal dose (LD_50_) of THC and OHC were estimated in mice on the basis of the Chemical-Test method of acute oral toxicity of the People’s Republic of China (PRC) National Standard (GB/T 21603-2008) and the food security of the PRC National Standard (GB 15193.3-2014). Briefly, for the acute toxicity test of THC, ICR mice fasted overnight were randomly divided into six groups, and each group consisted of 10 mice of either sex. Then the mice were administered orally with a graded doses of THC (0, 100, 316, 1000, 3160, and 10,000 mg/kg) in every test groups. Post treatment, the mice were provided with forage and water *ad libitum* and observed in an open-field environment for 14 consecutive days for any behavioral changes or mortality. The indications of clinical toxicity and number of deaths were recorded during this period. The acute toxicity test of OHC was the same as that of THC.

### Xylene-Induced Ear Edema in Mice

The xylene-induced ear edema test was carried out using the method described previously with some modifications (Li et al., 2016). In brief, on hour post treatment with THC, OHC, and CUR on the last day, all mice were smeared 20 μL of xylene on the anterior and posterior surface of the right ear to induce ear edema, while the left ear served as a control. Mice were sacrificed after another 60 min of xylene application. Ear disks of 8.0 mm in diameter were punched out and immediately weighed. The degree of ear edema was measured by the weight variation between the two ear disks of the same mouse.

### Acetic Acid-Induced Vascular Permeability in Mice

The acetic acid-induced vascular permeability test was carried out based on the previous method with certain modifications (Whittle, 1964). Briefly, after pre-treatment for 7 days, mice were intravenously injected with 1% Evans blue in physiological saline solution at 0.1 mL/10 g followed by an injection (i.p.) of 0.6% acetic acid at 0.1 mL/10 g body weight. Twenty minutes later, mice were sacrificed and the peritoneal cavity of each mouse was washed thrice with a total of 5 mL physiological saline. The collected liquid was centrifuged for 15 min at 550 × *g*, the supernatants were collected and measured at the wavelength (590 nm) by using an ultraviolet-visible spectrophotometry (Shimadzu Co., Ltd., Kyoto, Japan). The vascular permeability effects were evaluated based on the absorbency of dye.

### Carrageenan-Induced Mouse Paw Edema Model

The carrageenan-induced mouse hind paw edema test was employed using the method described anteriorly with some modifications (Albano et al., 2013). Briefly, after administration for 60 min, each animal except the intact control was injected with 25 μL of 1% freshly prepared carrageenan suspension at the plantar side of right hind paw. The paw edema values were evaluated before as the basal volume (V_o_) and 1, 2, 3, 4, 5, or 6 h as the pathological volume (V_i_) after carrageenan injection by the MK101CMP plethysmometer (Muromachi Kikai Co., Ltd., Japan). The percentage degree of swelling and inhibition of paw edema were calculated using the following formulas:

$$\%edema\;degree\;=\frac{V_{i}-V_{o}}{V_{o}}\times 100$$

$$\%inhibition\;=\frac{Mean\;edema\;(Carr)-Mean\;edema\;(drugs)}{Mean\;edema\;(Carr)}\times 100$$

Other mice were used to explore the molecular mechanism underlying the anti-inflammatory activities of THC and OHC. The mice were randomly assigned to six groups (*n* = 10): intact and vehicle controls (0.1% Tween-80); CUR control (100 mg/kg); THC group (40 mg/kg); OHC group (40 mg/kg); and Indo group (10 mg/kg). All mice were sacrificed by cervical dislocation at 4 h after carrageenan stimulation and the hind paws were then removed and instantly frozen (-80°C) until use. For biochemical analysis, the hind paws were placed in icy PBS (1:9, v/w) containing a protease inhibitor cocktail (Sigma-Aldrich), then homogenized using the Tissue Lyser II high-throughput tissue homogenization system (Qiagen Co., Ltd., Hilden, Germany). The homogenate was then incubated on ice for 15 min and centrifuged at 10000 × *g* for 15 min at 4°C, and the final supernatants were then obtained and stored at -80°C for investigation of the inflammatory factors.

### Measurement of IL-1β, IL-6, PGE_2_, and TNF-α by ELISA

The IL-1β, IL-6, PGE_2_ and TNF-α levels in the supernatants were measured using commercially available ELISA kit (R&D Systems, Abingdon, United Kingdom) following the manufacturer’s protocol. All the data were expressed as pg/mg tissue.

### Quantitative Real-Time RT-PCR (qRT-PCR) Analysis

Total RNA from the mouse paws prepared for the detection of COX-1 and COX-2 were isolated with TRIzol Reagent (Gibco, Grand Island, NY, United States) following the manufacturer’s instructions. From each sample, 1.5 μg RNA was employed to synthesize cDNA using Revert Aid First Strand cDNA Synthesis Kit (Thermo Fisher Scientific, Waltham, MA, United States) following the manufacturer’s protocol. RT-PCR was performed in a TaqMan fast universal PCR master mix kit (2 ×; Applied Biosystems, Foster City, CA, United States), under the Applied Biosystems Step-One Fast Real-Time PCR system, and the Sequence Detection Software 2.0 (Applied Biosystems, Inc., FosterCity, CA, United States) was used to analyze the results. The oligonucleotide primers for COX-1, designed from mouse (NM_008969.4), were CCTACAGCCCTTCAATGAATACC (forward) and GATGTCACCGTACAGCTCCTCC (reverse), respectively; for COX-2, designed from mouse (NC_010339), were ATAGACGAAATCAACAACCCCG (forward) and GGATTGGAAGTTCTATTGGCAG (reverse), respectively; for β-actin, designed from mouse (NM_007393.3), were GTGACGTTGACATCCGTAAAGA (forward) and GTAACAGTCCGCCTAGAAGCAC (reverse), respectively. The results were presented as the ratio of optimal density to β-actin.

### Western Blot Analysis

Western blot analysis was implemented to evaluate the effect of tested compounds on COX-1, COX-2, TAK1, p-TAK1, TAB1, IKKβ, p-IKKβ, IκBα, and p-IκBα protein expressions and the cytosol and nucleus expressions of NF-κB (p65). Briefly, the paw edema tissues were homogenized in lysis buffer containing a cocktail of phosphatase inhibitors and proteinase inhibitors. The homogenates were centrifuged at 12,000 × *g* for 20 min and equal protein extracts were separated using 10% SDS–PAGE gels and transferred to polyvinyl difluoride (PVDF) membranes. After the membranes were blocked with 5% skimmed milk at room temperature for 2 h in Tris-buffered saline-0.1% Tween-20 (TBS-T), they were then incubated with primary antibody (Cayman Co., United States). Sequentially the membranes were incubated with horseradish peroxidase-conjugated secondary antibody and detected by the enhanced chemiluminescence method (Amersham International plc., Buckinghamshire, United Kingdom).

### Statistical Analysis

All values in the figures and text were presented as means ± S.E.M. of triplicate experiments. *p* < 0.05 was deemed statistically significant. The results were analyzed using one-way analysis of variance (ANOVA) coupled with *post hoc* Dunnett’s test.

## Results

### Acute Toxicity

During the 14 days of behavioral observation, the animals, both in THC or OHC groups, showed no abnormal behavioral change, and no convulsions and deaths were observed. The LD_50_ values of graded doses of THC and OHC in mice were both greater than 10,000 mg/kg. Therefore, both THC and OHC possessed a wide margin of safety.

### Effects of THC and OHC on the Xylene-Induced Ear Edema in Mice

**FIGURE 2:**
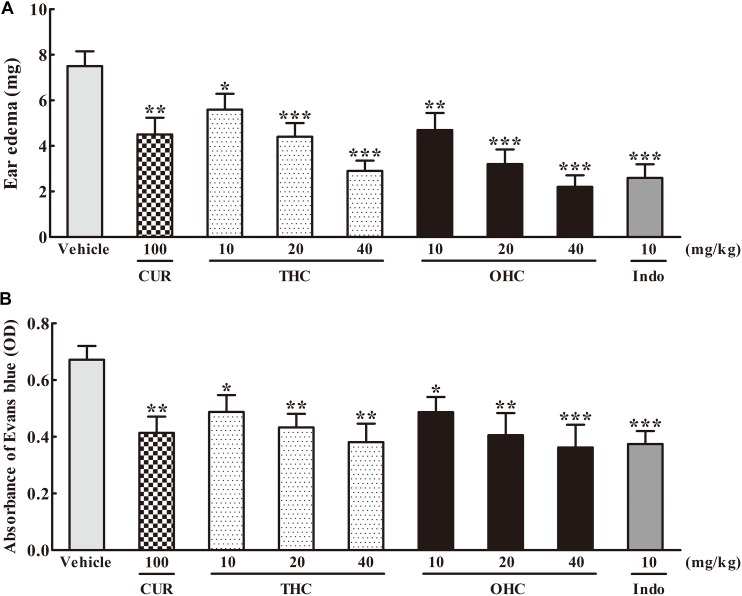
Effects of THC and OHC on the xylene-induced ear edema in mice **(A)** and acetic acid-induced vascular permeability in mice **(B)**. **(A)** The ear edema was assessed as the weight difference between the left and the right ear biopsies of the same animal. **(B)** The capillary permeability was represented as the amount of Evans blue extruded into peritoneal cavity, which was measured by the OD of the supernatant. Data were expressed as means ± S.E.M. (*n* = 10), ^∗^*p* < 0.05, ^∗∗^*p* < 0.01, ^∗∗∗^*p* < 0.001 vs. the vehicle control. Significant differences between groups were determined by ANOVA and Dunnett’s *post hoc* test.

As shown in **Figure 2A**, in comparison to the vehicle group, administration with THC (10, 20, and 40 mg/kg) or OHC (10, 20, and 40 mg/kg) displayed a dose-dependent attenuation on the ear edema induced by xylene. The suppression rate were 25.33% (*p* < 0.05), 41.33% (*p* < 0.001), and 61.33% (*p* < 0.001) for THC, respectively, and 37.33% (*p* < 0.01), 57.33% (*p* < 0.001), and 70.67% (*p* < 0.001) for OHC, respectively. On the other hand, CUR and Indo (positive control) dramatically inhibited the ear edema by 40% (*p* < 0.01) and 65.33% (*p* < 0.001), respectively.

### Effects of THC and OHC on the Acetic Acid-Induced Vascular Permeability in Mice

**Figure 2B** shows the effects of THC and OHC on the acetic acid-elicited vascular permeability in mice. In contrast to the vehicle group, administration with THC (10, 20, and 40 mg/kg) or OHC (10, 20, and 40 mg/kg) markedly and dose-dependently inhibited the vascular permeability. The suppressive ratios of THC and OHC at 40 mg/kg were up to 45.1% (*p* < 0.01) and 48.0% (*p* < 0.001), respectively, with the efficacy similar to that of Indo (46.2%, *p* < 0.001). The suppressive ratio of CUR for the acetic acid-induced vascular permeability was 40.0% (*p* < 0.01).

### Effects of THC and OHC on the Carrageenan-Induced Paw Edema in Mice

**FIGURE 3:**
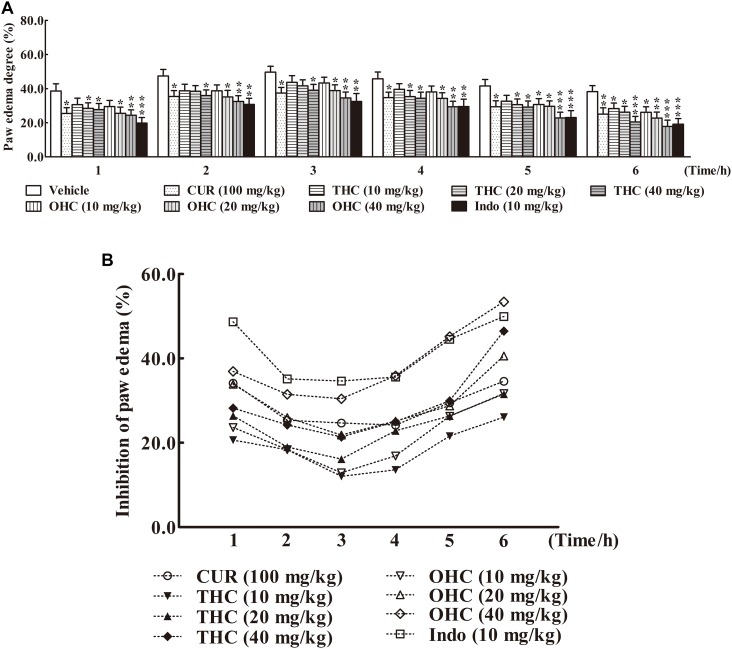
Effects of THC and OHC on the carrageenan-induced paw edema in mice. **(A)** Paw edema degree was measured using the toes swelling measuring instrument and was represented as the mean ± S.E.M. (*n* = 10). **(B)** Suppression of paw edema (%) was calculated as the ratio of the mean paw size increase of THC and OHC treatment groups (%) over the vehicle control group (%).vs. the vehicle control. Significant differences between groups were determined by ANOVA and Dunnett’s *post hoc* test. Data were expressed as means ± S.E.M. (*n* = 10), ^∗^*p* < 0.05, ^∗∗^*p* < 0.01, ^∗∗∗^*p* < 0.001 vs the vehicle control.

The carrageenan-induced paw edema in mice is a sensitive acute inflammation model to evaluate the inhibitory effect of candidate agents. After the carrageenan treatment, the volume of paw edema was substantially increased during the subsequent 6 h with a peak at 3 h (**Figure 3A**). In this experiment, administration with THC (10, 20, and 40 mg/kg) or OHC (10, 20, and 40 mg/kg) markedly attenuated the paw edema in a concentration-dependent manner from the 1st to the 6th h in comparison to the vehicle group (**Figures 3A,B**). Oral administration of THC (40 mg/kg) and OHC (40 mg/kg) exhibited superior effects in reducing the edema in parallel to the CUR control (100 mg/kg). Furthermore, in contrast to the positive control, pretreatment with OHC at the dosage of 40 mg/kg exhibited more potent inhibition in paw edema at 4th, 5th, and 6th h, with the inhibition ratios of 35.87% (*p* < 0.01), 45.16% (*p* < 0.001), and 53.41% (*p* < 0.001), respectively.

### Effects of THC and OHC on IL-1β, IL-6, TNF-α, and PGE_2_ Levels

**FIGURE 4:**
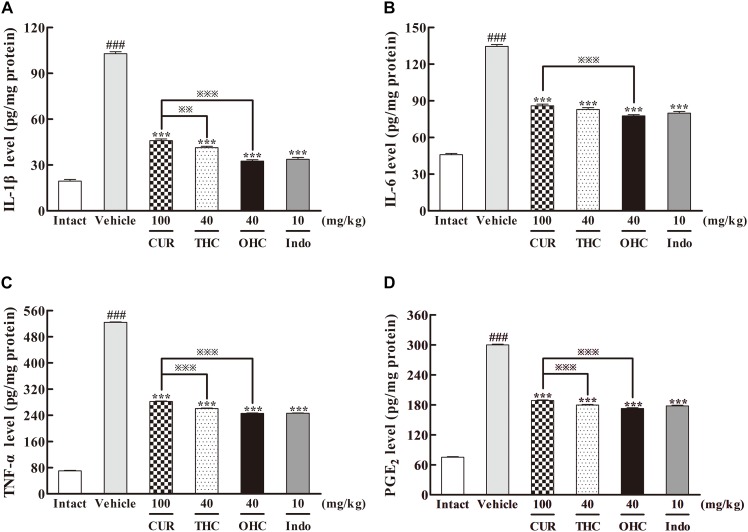
Effects of THC and OHC on the levels of IL-1β **(A)**, IL-6 **(B)**, TNF-α **(C)**, and PGE_2_ **(D)** in the carrageenan-induced mouse paw edema. After administration with THC (40 mg/kg, i.g.), OHC (40 mg/kg, i.g.) or CUR (100 mg/kg, i.g.) for 7 days, the hind paws were removed and placed in cold PBS (1:9, v/w). After homogenization and centrifugation, the resulting supernatants were collected and analyzed by ELISA kits. Data were expressed as means ± S.E.M. (*n* = 10), ^###^*p* < 0.001 vs. the intact control; ^∗∗∗^*p* < 0.001 vs. the vehicle control; ^
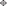
^*p* < 0.001 vs. the CUR group. Significant differences between groups were determined by ANOVA and Dunnett’s *post hoc* test.

To elucidate the underlying mechanisms of action, we examined the effects of THC and OHC at the dosage of 40 mg/kg on the carrageenan-induced pro-inflammatory mediators, including IL-1β, IL-6, TNF-α, and PGE_2_ by ELISA. As depicted in **Figure 4**, post carrageenan injection, the concentrations of IL-1β, IL-6, TNF-α, and PGE_2_ levels increased substantially in comparison to the intact control (all *p* < 0.001). In contrast, pretreatment with THC or OHC both significantly reduced the carrageenan-induced productions of IL-1β, IL-6, TNF-α, and PGE_2_, respectively (all *p* < 0.001), and the attenuating effect for both compounds was similar to that of the positive control. While CUR markedly inhibited the expressions of pro-inflammatory cytokines (all *p* < 0.001), it exhibited much lower inhibitory activities as compared to THC (IL-1β: *p* < 0.01, TNF-α and PGE_2_: all *p* < 0.001) and OHC (IL-1β, IL-6, TNF-α, and PGE_2_: all *p* < 0.001), indicating that the metabolites of CUR might be more potent than CUR in inhibiting the carrageenan-induced pro-inflammatory cytokines.

### Effects of THC and OHC on the Gene and Protein Levels of COX-1 and COX-2

**FIGURE 5:**
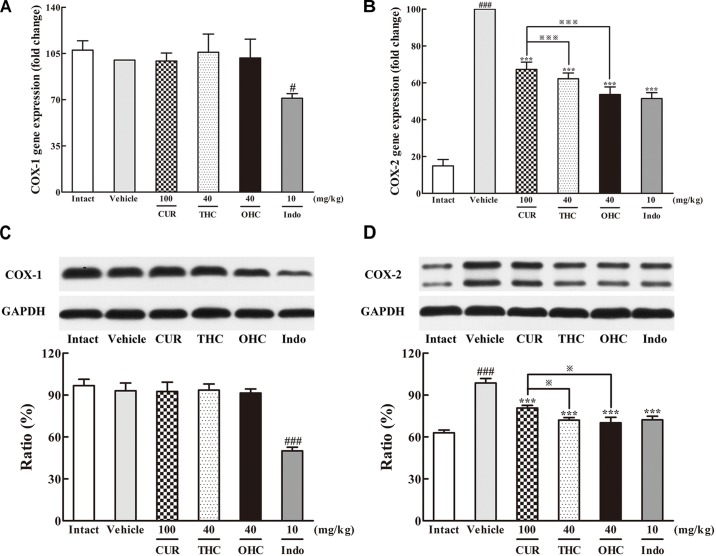
Effects of THC and OHC on the mRNA **(A,B)** and protein **(C,D)** expressions of COX-1 and COX-2 in the carrageenan-induced mouse paw edema. After administration with THC (40 mg/kg, i.g.), OHC (40 mg/kg, i.g.) or CUR (100 mg/kg, i.g.) for 7 days, the hind paws were removed and isolated with TRIzol Reagent for analysis of mRNA expression of COX-1 and COX-2. Other hind paws were removed and homogenized in the lysis buffer, and then the protein extracts of hind paws were transferred to polyvinyl difluoride (PVDF) membranes and analyzed by Western blot. β-Actin was employed as the control for PCR. GAPDH was employed as loading control for Western blot. Data were expressed as means ± S.E.M. (*n* = 3), ^#^*p* < 0.05, ^###^*p* < 0.001 vs. the intact control; ^∗∗∗^*p* < 0.001 vs. the vehicle control; ^
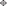
^*p* < 0.001 vs. the CUR group. Significant differences between groups were determined by ANOVA and Dunnett’s *post hoc* test.

The results of the respective inhibitory activities of THC and OHC on the production of COX-1 and COX-2 in the carrageenan-induced paw edema were shown in **Figure 5**. In the intact control, the gene and protein levels of COX-1 were both widely expressed *in vivo*, while the expression of COX-2 was much lower than that of COX-1. However, upon the carrageenan treatment, both the gene and protein expression of COX-2 were dramatically increased when compared with the intact control. In contrast, COX-1 expression barely changed upon carrageenan treatment. Oral administration of THC or OHC obviously down-regulated the gene and protein levels of COX-2 (all *p* < 0.001) but exerted no significant influence on the COX-1 expression. The positive control was able to significantly inhibit the expression of COX-1 (gene level: *p* < 0.05, protein level: *p* < 0.001) and COX-2 (all *p* < 0.001), respectively. The results indicate that THC and OHC might selectively and markedly suppress the COX-2 expression without obviously affecting the COX-1 expression. Moreover, the results implied that treatment with THC and OHC were more effective than CUR in inhibiting the expression of COX-2 (gene level: both *p* < 0.001, protein level: both *p* < 0.05).

### Effects of THC and OHC on the Interaction Between TAK1 and TAB1 Protein Expressions

**FIGURE 6:**
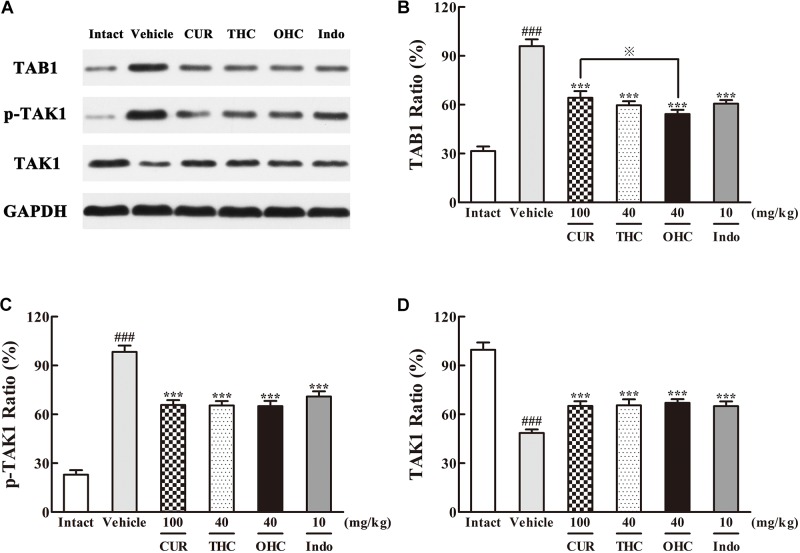
Effects of **(A)** Representative lanes of TAB1, p-TAK1, and TAK1. THC and OHC on TAB1 **(B)**, p-TAK1 **(C)**, and TAK1 **(D)** protein expressions in the carrageenan-induced mouse paw edema. After administration with THC (40 mg/kg, i.g.), OHC (40 mg/kg, i.g.) or CUR (100 mg/kg, i.g.) for 7 days, the hind paws were removed and homogenized in the lysis buffer, and then the protein extracts of hind paws were transferred to polyvinyl difluoride (PVDF) membranes and analyzed by Western blot. GAPDH was employed as loading control. Data were expressed as means ± S.E.M. (*n* = 3), ^###^*p* < 0.001 vs. the intact control; ^∗∗∗^*p* < 0.001 vs. the vehicle control; ^
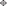
^*p* < 0.001 vs. the CUR group. Significant differences between groups were determined by ANOVA and Dunnett’s *post hoc* test.

The different influences of THC and OHC on the production of TAK1 and TAB1 in the carrageenan-induced paw edema were shown in **Figure 6**. After the carrageenan treatment, TAB1 protein expression in the vehicle group was much obvious than that in the intact control (**Figure 6B**, *p* < 0.001); besides, the increased protein level of p-TAK1 and decreased protein level of TAK1 were also indicative of the significant TAK1 phosphorylation induced by the carrageenan-treatment (**Figures 6C,D**, all *p* < 0.001). By contrast, oral administration of THC or OHC significantly inhibited the TAK1 phosphorylation (*p* < 0.001). Meanwhile, binding of TAK1 to TAB1 was also substantially reduced after treatment with THC or OHC, and the binding effects of THC and OHC were analogous to that of the positive control, with a much lower protein levels of p-TAK1 and higher protein levels of TAK1 (**Figure 6**, all *p* < 0.001). In addition, our results also showed that OHC exhibited more potent inhibitory effect on the protein expression of TAB1 than CUR (**Figure 6B**, *p* < 0.05).

### Effects of THC and OHC on the Interaction Between NF-κB and IKKβ Protein Expressions

**FIGURE 7:**
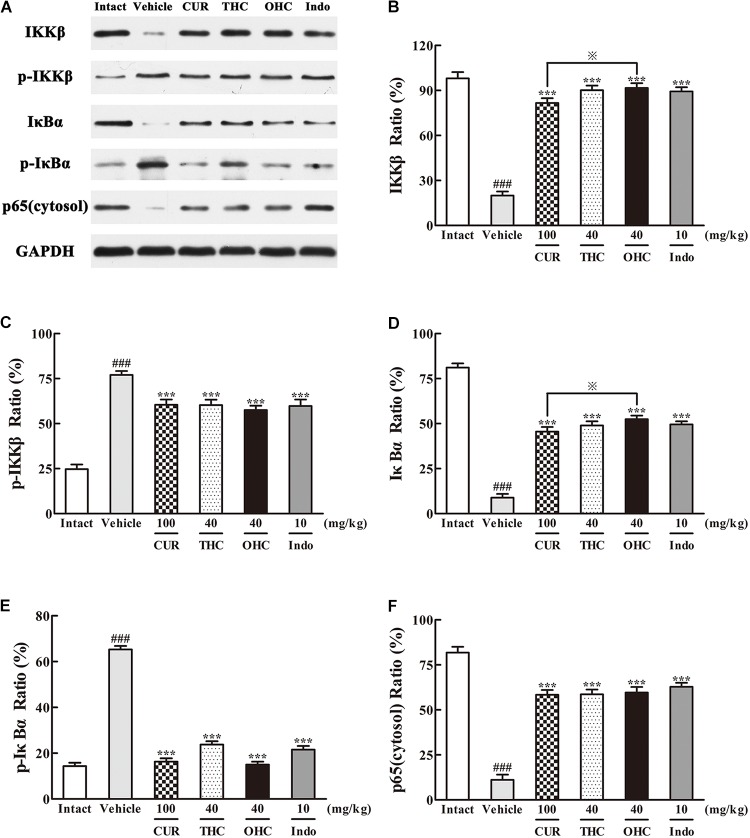
Effects of **(A)** Representative lanes of IKKβ, p-IKKβ, IκBα, p-IκBα and p65 (cytosol). THC and OHC on IKKβ **(B)**, p-IKKβ **(C)**, IκBα **(D)**, p-IκBα **(E)**, and p65 (cytosol) **(F)** protein expressions in the carrageenan-induced mouse paw edema. After administration with THC (40 mg/kg, i.g.), OHC (40 mg/kg, i.g.) or CUR (100 mg/kg, i.g.) for 7 days, the hind paws were removed and homogenized in the lysis buffer, and then the protein extracts of hind paws were transferred to polyvinyl difluoride (PVDF) membranes and analyzed by Western blot. GAPDH was employed as loading control. Data were expressed as means ± S.E.M. (*n* = 3), ^###^*p* < 0.001 vs. the intact control; ^∗∗∗^*p* < 0.001 vs. the vehicle control; ^
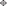
^*p* < 0.001 vs. the CUR group. Significant differences between groups were determined by ANOVA and Dunnett’s *post hoc* test.

**FIGURE 8:**
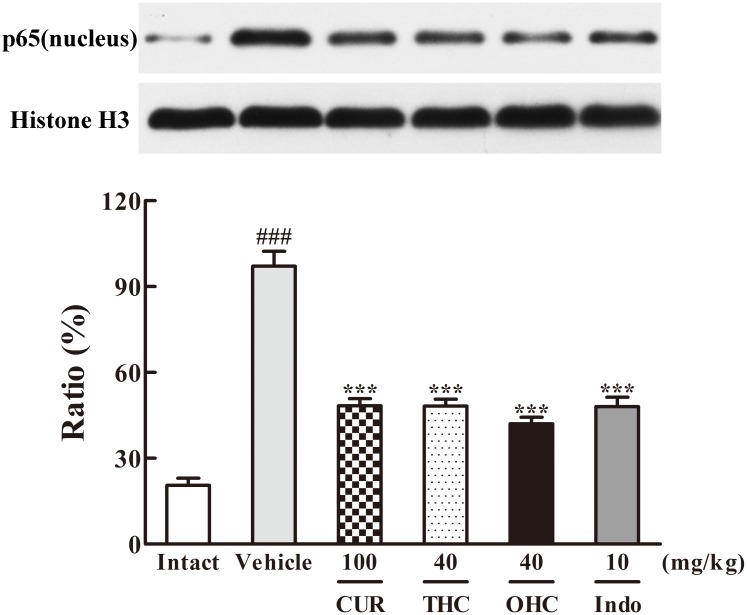
Effects of THC and OHC on p65 (nucleus) protein expression in the carrageenan-induced mouse paw edema. After administration with THC (40 mg/kg, i.g.), OHC (40 mg/kg, i.g.) or CUR (100 mg/kg, i.g.) for 7 days, the hind paws were removed and homogenized in the lysis buffer, and then the protein extracts of hind paws were transferred to PVDF membranes and analyzed by Western blot. Histone H3 was employed as loading control. Data were expressed as means ± S.E.M. (*n* = 3), ^###^*p* < 0.001 vs. the intact control; ^∗∗∗^*p* < 0.001 vs. the vehicle control. Significant differences between groups were determined by ANOVA and Dunnett’s *post hoc* test.

Western blot analysis of the IKKβ phosphorylation and NF-κB expression was performed to evaluate the cellular mechanisms. As shown in **Figures 7, 8**, treatment with carrageenan led to a marked attenuation in IKKβ levels and a significant increase in p-IKKβ levels when compared to the intact control (**Figures 7B,C**, all *p* < 0.001). In contrast, THC and OHC treatment markedly prevented the carrageenan-induced IKKβ phosphorylation (all *p* < 0.001).

Since IKKβ is the upstream kinases of IκBα in the NF-κB signaling pathway, we then investigated the effects of THC or OHC on the carrageenan-induced IκBα and NF-κB activation in paw edema. It was found that IKKβ phosphorylation led to obvious IκBα degradation and p-IκBα expression in the vehicle control, subsequently freed NF-κB (p65) and allowed it to be translocated to the nucleus. However, administration with THC or OHC significantly suppressed the carrageenan-induced degradation and phosphorylation of IκBα, and markedly inhibited the translocation of p65 from the cytosol to the nucleus in parallel to the vehicle control (**Figures 7, 8**, all *p* < 0.001). Furthermore, oral administration of OHC was more effective than CUR in increasing IKKβ and IκBα levels. These findings unambiguously suggest that THC and OHC were able to prevent NF-κB activation by inhibiting the TAK1/ IKKβ0/ IκBα pathway in the carrageenan-elicited paw edema in mice.

## Discussion

To date, CUR was regarded as a valuable preventive and therapeutic agent against a variety of diseases. However, despite its good safety and efficacy profiles, CUR has not yet been approved in the clinical application mainly due to its rapid metabolism and poor absorption in the body (Anand et al., 2007). The poor systemic bioavailability restricts its clinical development. Over the past few years, researchers have turned their attention to its reductive metabolites in an attempt to figure out why CUR possesses remarkable biological effects in spite of its poor absorption. As the two major metabolites of CUR, THC, and OHC are known to be reduced by endogenous reductase system. They have been reported to exhibit potent anti-inflammatory effect *in vitro* (Pan et al., 2000). However, evidence for the anti-inflammatory effect of THC and OHC *in vivo* is still lacking. THC has been reported to be more stable than CUR in saline and in phosphate buffer (Yodkeeree et al., 2008), and this property renders it worthwhile for further biological investigation. Furthermore, as the ultimate reduced metabolite of CUR *in vivo*, OHC possesses the most active reducibility.

Inflammation is a defense mechanism which plays an important role in tissue repair and elimination of injurious stimuli. However, inflammatory response is a double-edged sword. Persistent inflammatory reaction would have insidious effects and could be harmful to the tissues (Shin et al., 2008). Therefore, research and development on anti-inflammatory drugs has always been an important scientific pursuit. Historically, traditional non-steroidal anti-inflammatory drugs (NSAIDs) have been applied clinically for a wide range of inflammatory diseases such as multiple sclerosis and rheumatoid arthritis (RA) (Gautam and Jachak, 2009). However, these agents can cause severe adverse effects such as gastric ulcer and bleeding (Wallace, 2001). Hence, it is extraordinarily desirable to explore safe and efficient therapeutic agents for inflammatory conditions. Recent decades have witnessed many endeavors on development of new anti-inflammatory drugs from natural products and herbal medicines using inflammatory animal models, including mouse ear edema induced by xylene, mouse vascular permeability elicited by acetic and mouse paw edema provoked by carrageenan.

The xylene-elicited mice ear edema is one of the typical models for acute inflammation, and is usually employed to screen the *in vivo* anti-inflammatory effect of candidates in mice. Xylene induces the release of inflammatory mediators, causing inflammation edema (Cheng et al., 2016). The acetic acid-provoked vascular permeability assay is another well-established murine model applied to assess the anti-inflammatory potential of candidates. Intraperitoneal injection of acetic acid into mice could cause the release of inflammatory mediators, leading to the increase of vascular permeability, edema and eventual tissue injuries. Carrageenan-induced paw edema is a standard, nonimmune and highly reproducible acute inflammatory model, which is commonly employed to explore the anti-inflammatory activity and underlying mechanism of candidates (Archer et al., 2015). Injection of carrageenan provokes the releases of inflammatory cytokines, extravasation of fluid and proteins, and the accumulation of leukocyte, resulting in the formation of paw edema. Among these three common inflammatory models, the carrageenan-induced mouse paw edema is considered to be a standard model to evaluate *in vivo* action mechanism of new agents during the inflammatory process (Impellizzeri et al., 2011). The present study aims to provide the first evidence on whether THC and OHC could attenuate inflammatory response and to assess the underlying molecular mechanism of THC and OHC using the carrageenan-induced mouse paw edema model.

The result of acute toxicology test indicated that THC and OHC possessed better safety profile than that of CUR. Further attempt was made to compare the anti-inflammatory effect of THC, OHC, and CUR *in vivo*. The xylene-elicited ear edema and acetic acid-elicited vascular permeability are two classical animal models for primarily evaluating the anti-inflammatory potential of candidates. In these assays, administration with THC (10, 20, and 40 mg/kg) or OHC (10, 20, and 40 mg/kg) markedly suppressed the formation of xylene-elicited edema and inhibited the acetic acid-induced Evans blue dye leakage in peritoneal cavity in a concentration-dependent manner, suggesting the anti-inflammatory action of THC and OHC.

To further validate the anti-inflammatory effect of THC and OHC *in vivo*, the carrageenan-elicited paw edema model was employed. Our experimental data showed that treatment with THC (10, 20, and 40 mg/kg) or OHC (10, 20, and 40 mg/kg) dose-dependently abrogated the formation of the paw edema when compared to the vehicle control after carrageenan treatment, indicating that THC and OHC were capable of preventing the carrageenan-induced acute inflammation.

It has been known that carrageenan can induce the peripheral release of IL-1β, IL-6, TNF-α and enhances the PGE_2_ synthesis in tissues (Omote et al., 2001). IL-1β, TNF-α, and IL-6 are the essential cytokines in the early pathogenesis of immune response to promote the expressions of inflammation-related mediators such as prostaglandins, leading to the accentuation of inflammation. As one of the most important prostaglandins, PGE_2_, which is synthesized and catalyzed by COX-2, plays a central role in pain, fever and inflammatory diseases (Tsatsanis et al., 2006). Therefore, it can be an efficient strategy to deal with various inflammatory diseases by developing new anti-inflammatory drugs that could directly inhibit these pro-inflammatory cytokines. In particular, selective COX-2 inhibitors have received enthusiastic attention because they could avoid the side effects of COX-1 inhibition commonly brought about by traditional NSAIDs. NSAIDs have served as the primary agents for inflammation in clinical practice for a long time. However, they can cause serious side effects such as ulcers and bleeding in the gastrointestinal tract (Radi and Khan, 2006). Thus, selective COX-2 inhibitors are greatly needed for inflammation therapy with regard to safety issues related to potential COX-1 inhibition. In the present study, THC and OHC suppressed the tissue levels of IL-1β, IL-6, TNF-α, and PGE_2_, indicating that THC and OHC could alleviate acute inflammation by mitigating the production of pro-inflammatory mediators. Moreover, both THC and OHC significantly inhibited the expression of COX-2 in parallel to the vehicle control, while it exerted no obvious influence on the expression of COX-1. Additionally, THC and OHC showed superior effects in inhibiting the levels of pro-inflammatory mediators and suppressing the expression of COX-2 when compared with CUR, suggesting that THC and OHC might possess better effect than CUR in selectively inhibiting the COX-2 activity.

Massive evidences have confirmed that NF-κB activation could be triggered by overexpression of TAK1 and its adapter protein TAB1 (Sakurai et al., 1998). NF-κB activation could eventually lead to a promotion of pro-inflammatory cytokines like IL-6 and TNF-α. Therefore, TAK1 is defined as a crucial kinase for the activation of NF-κB pathway. After activated by p-TAK1, the IKK complex is then phosphorylated, subsequently leading to the phosphorylation and degradation of IκBs. Phosphorylation of IκB would further induce the translocation of NF-κB and eventually activates a variety of downstream target genes. Our data clearly showed that THC and OHC treatment significantly down-regulated the interaction between TAK1 and TAB1 as induced by carrageenan treatment, suppressed TAK1, IKKβ, and IκBα phosphorylation, and prevented the translocation of NF-κB (p65) from the cytosol to the nucleus in comparison to the vehicle control. These results implied that the suppression of NF-κB activation by THC and OHC might be related to the inhibition of TAK1 phosphorylation and stabilization of IκBα activation. Furthermore, OHC markedly promoted the expression of IKKβ and IκBα, reduced the expression of TAB1 as compared with CUR, indicating that OHC supposedly played an important role in the CUR-induced anti-inflammatory activities.

Structurally, THC and OHC share analogous structure when compare to CUR but differ in that CUR harbors α, β dienes, while the THC and OHC have more phenolic groups (Zhao et al., 2015). It has been known that CUR has poor absorption and is very unstable in aqueous solution (Anand et al., 2007). Ascribed to these drawbacks, the clinical application of CUR has so far been limited. Moreover, the hydrophobic nature of CUR contributes to its poor bioavailability. In contrast, both THC and OHC are more soluble and stable compared with CUR, rendering them more likely to be applied in the clinical practice. In the present work, we proposed that the distinctions between the anti-inflammatory activities of THC, OHC and CUR *in vivo* may be attributed to their structural discrepancies. Hydrogenation at conjugated double bonds of the pentadiene and β-diketone of CUR and conversion of it to THC or OHC might remarkably enhance the anti-inflammatory effects in the mouse paw edema elicited by carrageenan.

Taken together, this was the first investigation to explore the anti-inflammatory effects of THC and OHC, two of the important metabolites of CUR, *in vivo*. It was found that THC and OHC treatment exhibited potent anti-inflammatory effects, and the mechanism was intimately associated with the suppression of NF-κB signaling pathways through TAK1 inactivation, and subsequent inhibition of COX-2 activity and other inflammatory mediators. In addition, our experimental findings also illustrated that the anti-inflammatory activities of THC and OHC were both more pronounced than CUR, indicating that THC or OHC might be the significant bioactive anti-inflammatory forms of CUR *in vivo*. Our results strongly indicate that THC or OHC are promising new chemical entities for further development into potent anti-inflammatory therapeutics.

## Author Contributions

Idea of the research and writing assistance were provided by Z-XL and Z-RS. J-HX, Y-FX, and Z-QL contributed to the experimental performance and data collection. Y-HL and W-HL carried out the data evaluation. Z-BZ and D-DL participated in the experimental performance and coordination and drafted this manuscript. Manuscript proofreading was performed by J-NC and X-PL. All authors read and approved the final manuscript.

## Conflict of Interest Statement

The authors declare that the research was conducted in the absence of any commercial or financial relationships that could be construed as a potential conflict of interest. The reviewer GO and handling Editor declared their shared affiliation at the time of review.

## Supplementary Material

FIGURE S1 Dose-response results for the effects of THC and OHC on the xyleneinduced ear edema in mice **(A)** and acetic acid-induced vascular permeability in mice **(B)** in our prior trial. Data are expressed as means ± S.E.M. (*n* = 10), ^∗^*p* < 0.05, ^∗∗^*p* < 0.01, ^∗∗∗^*p* < 0.001vs the vehicle control. Significant differences between groups were determined by ANOVA and Dunnett’s *post hoc* test.

## References

[B1] Agrawal D. K. Mishra P. K. (2010). Curcumin and its analogues: potential anticancer agents. *Med. Res. Rev.* 30 818–860. 10.1002/med.20188 20027668

[B2] Akaogi J. Nozaki T. Satoh M. Yamada H. (2006). Role of PGE2 and EP receptors in the pathogenesis of rheumatoid arthritis and as a novel therapeutic strategy. *Endocr. Metab. Immune Disord. Drug Targets* 6 383–394. 10.2174/187153006779025711 17214584

[B3] Albano M. N. da Silveira M. R. Danielski L. G. Florentino D. Petronilho F. Piovezan A. P. (2013). Anti-inflammatory and antioxidant properties of hydroalcoholic crude extract from *Casearia sylvestris* Sw. (Salicaceae). *J. Ethnopharmacol.* 147 612–617. 10.1016/j.jep.2013.03.049 23542040

[B4] Anand P. Kunnumakkara A. B. Newman R. A. Aggarwal B. B. (2007). Bioavailability of curcumin: problems and promises. *Mol. Pharm.* 4 807–818. 10.1021/mp700113r 17999464

[B5] Archer A. C. Muthukumar S. P. Halami P. M. (2015). Anti-inflammatory potential of probiotic *Lactobacillus* spp. On carrageenan induced paw edema in Wistar rats. *Int. J. Biol. Macromol.* 81 530–537. 10.1016/j.ijbiomac.2015.08.044 26314910

[B6] Cheng J. Ma T. Liu W. Wang H. Jiang J. Wei Y. (2016). In in vivo evaluation of the anti-inflammatory and analgesic activities of compound Muniziqi granule in experimental animal models. *BMC Complement. Altern. Med.* 16:20. 10.1186/s12906-016-0999-y 26800679 PMC4722770

[B7] Doyle S. L. O’Neill L. A. (2006). Toll-like receptors: from the discovery of NFkappaB to new insights into transcriptional regulations in innate immunity. *Biochem. Pharmacol.* 72 1102–1113. 10.1016/j.bcp.2006.07.010 16930560

[B8] Gautam R. Jachak S. M. (2009). Recent developments in anti-inflammatory natural products. *Med. Res. Rev.* 29 767–820. 10.1002/med.20156 19378317

[B9] Hacker H. Karin M. (2006). Regulation and function of IKK and IKK-related kinases. *Sci. STKE* 2006:re13. 10.1126/stke.3572006re13 17047224

[B10] Huang G. J. Huang S. S. Lin S. S. Shao Y. Y. Chen C. C. Hou W. C. (2010). Analgesic effects and the mechanisms of anti-inflammation of ergostatrien-3beta-ol from *Antrodia camphorata* submerged whole broth in mice. *J. Agric. Food Chem.* 58 7445–7452. 10.1021/jf1013764 20507140

[B11] Impellizzeri D. Esposito E. Mazzon E. Paterniti I. Di Paola R. Bramanti P. (2011). Effect of apocynin, a NADPH oxidase inhibitor, on acute lung inflammation. *Biochem. Pharmacol.* 81 636–648. 10.1016/j.bcp.2010.12.006 21147071

[B12] Ivanov A. I. Pero R. S. Scheck A. C. Romanovsky A. A. (2002). Prostaglandin E(2)-synthesizing enzymes in fever: differential transcriptional regulation. *Am. J. Physiol. Regul. Integr. Comp. Physiol.* 283 R1104–R1117. 10.1152/ajpregu.00347.2002 12376404

[B13] Lee S. L. Huang W. J. Lin W. W. Lee S. S. Chen C. H. (2005). Preparation and anti-inflammatory activities of diarylheptanoid and diarylheptylamine analogs. *Bioorg. Med. Chem.* 13 6175–6181. 10.1016/j.bmc.2005.06.058 16084726

[B14] Li X. J. Yang Y. J. Li Y. S. Zhang W. K. Tang H. B. (2016). Alpha-Pinene, linalool, and 1-octanol contribute to the topical anti-inflammatory and analgesic activities of frankincense by inhibiting COX-2. *J. Ethnopharmacol.* 179 22–26. 10.1016/j.jep.2015.12.039 26721216

[B15] Monaco C. Andreakos E. Kiriakidis S. Feldmann M. Paleolog E. (2004). T-cell-mediated signalling in immune, inflammatory and angiogenic processes: the cascade of events leading to inflammatory diseases. *Curr. Drug Targets Inflamm. Allergy* 3 35–42. 10.2174/1568010043483881 15032640

[B16] Omote K. Hazama K. Kawamata T. Kawamata M. Nakayaka Y. Toriyabe M. (2001). Peripheral nitric oxide in carrageenan-induced inflammation. *Brain Res.* 912 171–175. 10.1016/S0006-8993(01)02733-0 11532433

[B17] Pan M. H. Lin-Shiau S. Y. Lin J. K. (2000). Comparative studies on the suppression of nitric oxide synthase by curcumin and its hydrogenated metabolites through down-regulation of IkappaB kinase and NFkappaB activation in macrophages. *Biochem. Pharmacol.* 60 1665–1676. 10.1016/S0006-2952(00)00489-5 11077049

[B18] Patrignani P. Panara M. R. Greco A. Fusco O. Natoli C. Iacobelli S. (1994). Biochemical and pharmacological characterization of the cyclooxygenase activity of human blood prostaglandin endoperoxide synthases. *J. Pharmacol. Exp. Ther.* 271 1705–1712. 7996488

[B19] Prickett T. D. Ninomiya-Tsuji J. Broglie P. Muratore-Schroeder T. L. Shabanowitz J. Hunt D. F. (2008). TAB4 stimulates TAK1-TAB1 phosphorylation and binds polyubiquitin to direct signaling to NF-kappaB. *J. Biol. Chem.* 283 19245–19254. 10.1074/jbc.M800943200 18456659 PMC2443674

[B20] Radi Z. A. Khan N. K. (2006). Effects of cyclooxygenase inhibition on the gastrointestinal tract. *Exp. Toxicol. Pathol.* 58 163–173. 10.1016/j.etp.2006.06.004 16859903

[B21] Roth G. J. Stanford N. Majerus P. W. (1975). Acetylation of prostaglandin synthase by aspirin. *Proc. Natl. Acad. Sci. U.S.A.* 72 3073–3076. 10.1073/pnas.72.8.3073 810797 PMC432922

[B22] Sakurai H. Shigemori N. Hasegawa K. Sugita T. (1998). TGF-beta-activated kinase 1 stimulates NF-kappa B activation by an NF-kappa B-inducing kinase-independent mechanism. *Biochem. Biophys. Res. Commun.* 243 545–549. 10.1006/bbrc.1998.8124 9480845

[B23] Sandur S. K. Pandey M. K. Sung B. Ahn K. S. Murakami A. Sethi G. (2007). Curcumin, demethoxycurcumin, bisdemethoxycurcumin, tetrahydrocurcumin and turmerones differentially regulate anti-inflammatory and anti-proliferative responses through a ROS-independent mechanism. *Carcinogenesis* 28 1765–1773. 10.1093/carcin/bgm123 17522064

[B24] Santos L. A. Ribeiro E. L. Barbosa K. P. Fragoso I. T. Gomes F. O. Donato M. A. (2014). Diethylcarbamazine inhibits NF-kappaB activation in acute lung injury induced by carrageenan in mice. *Int. Immunopharmacol.* 23 153–162. 10.1016/j.intimp.2014.08.017 25175917

[B25] Sharma R. A. McLelland H. R. Hill K. A. Ireson C. R. Euden S. A. Manson M. M. (2001). Pharmacodynamic and pharmacokinetic study of oral Curcuma extract in patients with colorectal cancer. *Clin. Cancer Res.* 7 1894–1900. 11448902

[B26] Shim J. H. Xiao C. Paschal A. E. Bailey S. T. Rao P. Hayden M. S. (2005). TAK1, but not TAB1 or TAB2, plays an essential role in multiple signaling pathways in vivo. *Genes Dev.* 19 2668–2681. 10.1101/gad.1360605 16260493 PMC1283960

[B27] Shin E. M. Zhou H. Y. Guo L. Y. Kim J. A. Lee S. H. Merfort I. (2008). Anti-inflammatory effects of glycyrol isolated from *Glycyrrhiza uralensis* in LPS-stimulated RAW264.7 macrophages. *Int. Immunopharmacol.* 8 1524–1532. 10.1016/j.intimp.2008.06.008 18621150

[B28] Tsatsanis C. Androulidaki A. Venihaki M. Margioris A. N. (2006). Signalling networks regulating cyclooxygenase-2. *Int. J. Biochem. Cell Biol.* 38 1654–1661. 10.1016/j.biocel.2006.03.021 16713323

[B29] Vane J. R. (1971). Inhibition of prostaglandin synthesis as a mechanism of action for aspirin-like drugs. *Nat. New Biol.* 231 232–235. 10.1038/newbio231232a0 5284360

[B30] Wallace J. L. (2001). Pathogenesis of NSAID-induced gastroduodenal mucosal injury. *Best Pract. Res. Clin. Gastroenterol.* 15 691–703. 10.1053/bega.2001.0229 11566035

[B31] Whittle B. A. (1964). The use of changes in capillary permeability in mice to distinguish between narcotic and nonnarcotic alalgesics. *Br. J. Pharmacol. Chemother.* 22 246–253. 10.1111/j.1476-5381.1964.tb02030.x 14190460 PMC1703970

[B32] Yamaguchi K. Shirakabe K. Shibuya H. Irie K. Oishi I. Ueno N. (1995). Identification of a member of the MAPKKK family as a potential mediator of TGF-beta signal transduction. *Science* 270 2008–2011. 10.1126/science.270.5244.2008 8533096

[B33] Yodkeeree S. Garbisa S. Limtrakul P. (2008). Tetrahydrocurcumin inhibits HT1080 cell migration and invasion via downregulation of MMPs and uPA. *Acta Pharmacol. Sin.* 29 853–860. 10.1111/j.1745-7254.2008.00792.x 18565284

[B34] Zhang Z. Luo D. Xie J. Lin G. Zhou J. Liu W. (2018). Octahydrocurcumin, a final hydrogenated metabolite of curcumin, possesses superior anti-tumor activity through induction of cellular apoptosis. *Food Fusnct.* 9 2005–2014. 10.1039/c7fo02048a 29616245

[B35] Zhao F. Gong Y. Hu Y. Lu M. Wang J. Dong J. (2015). Curcumin and its major metabolites inhibit the inflammatory response induced by lipopolysaccharide: translocation of nuclear factor-kappaB as potential target. *Mol. Med. Rep.* 11 3087–3093. 10.3892/mmr.2014.3079 25502175

[B36] Zhongfa L. Chiu M. Wang J. Chen W. Yen W. Fan-Havard P. (2012). Enhancement of curcumin oral absorption and pharmacokinetics of curcuminoids and curcumin metabolites in mice. *Cancer Chemother. Pharmacol.* 69 679–689. 10.1007/s00280-011-1749-y 21968952 PMC3368221

