# Supplementary figures and images for: Curcumin’s Metabolites, Tetrahydrocurcumin and Octahydrocurcumin, Possess Superior Anti-inflammatory Effects in vivo Through Suppression of TAK1-NF-κB Pathway
Source: Front Pharmacol. 2018 Oct 17;9:1181. doi: 10.3389/fphar.2018.01181 (PMC6199526; doi:10.3389/fphar.2018.01181)

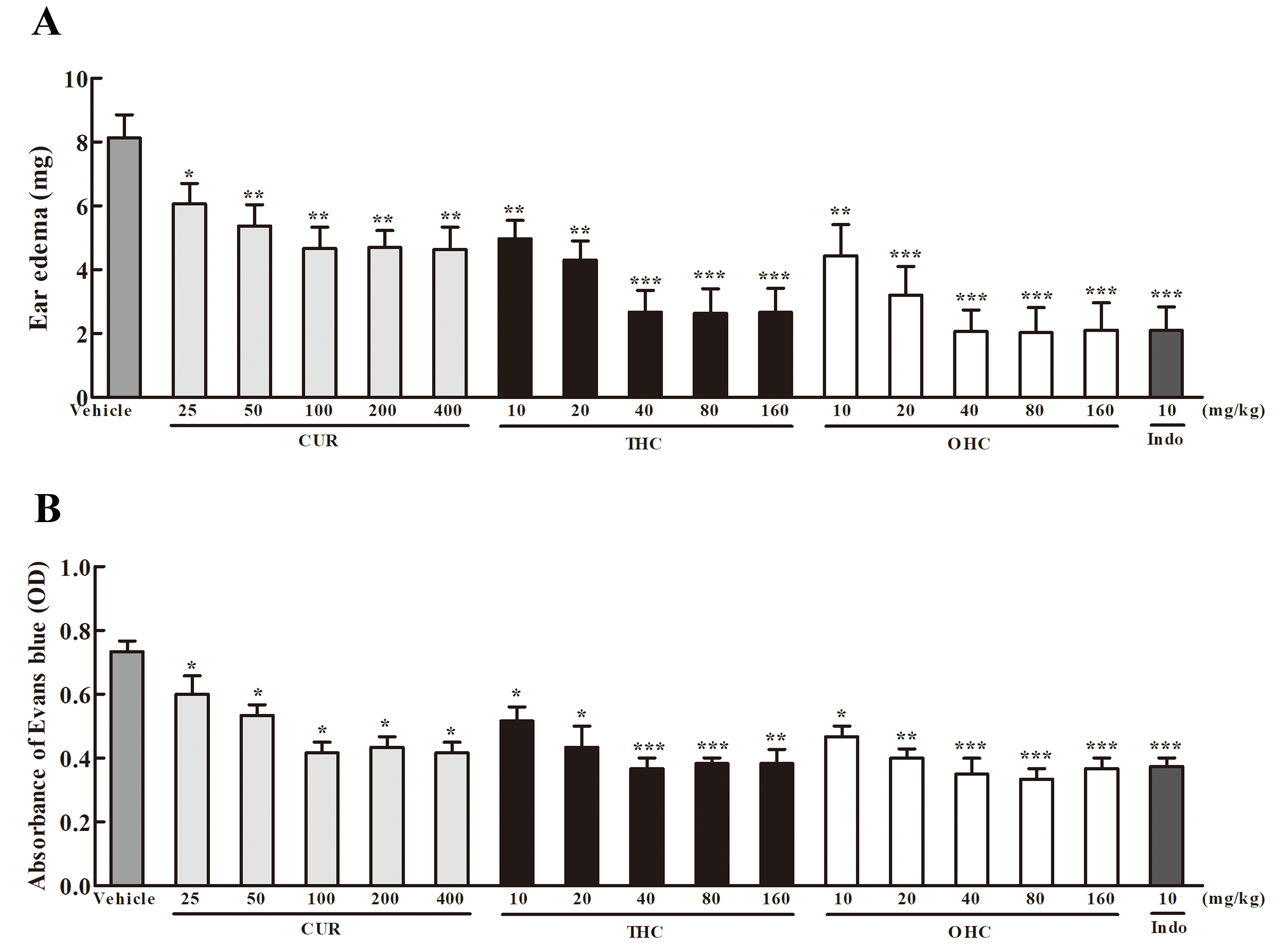

Supplement: FIGURE S1 — Dose-response results for the effects of THC and OHC on the xyleneinduced ear edema in mice (A) and acetic acid-induced vascular permeability in mice (B) in our prior trial. Data are expressed as means ± S.E.M. (n = 10), ∗p < 0.05, ∗∗p < 0.01, ∗∗∗p < 0.001vs the vehicle control. Significant differences between groups were determined by ANOVA and Dunnett’s post hoc test. [file Image_1.TIF]
